# Effect of Self-Adhesive and Separate Etch Adhesive Dual Cure Resin Cements on the Bond Strength of Fiber Post to Dentin at Different Parts of the Root

**Published:** 2017-05

**Authors:** Ehsan Mohamadian Amiri, Fariba Balouch, Faezeh Atri

**Affiliations:** 1 Postgraduate Student, Department of Prosthodontics, School of Dentistry, Babol University of Medical Sciences, Babol, Iran; 2 Assistant Professor, Department of Prosthodontics, Dental Branch, Islamic Azad University, Tehran, Iran; 3 Assistant Professor, Department of Prosthodontics, School of Dentistry, Tehran University of Medical Sciences, Tehran, Iran

**Keywords:** Post and Core Technique, Resin Cement, Dental Bonding, Shear Strength

## Abstract

**Objectives:**

Bonding of fiber posts to intracanal dentin is challenging in the clinical setting. This study aimed to compare the effect of self-adhesive and separate etch adhesive dual cure resin cements on the bond strength of fiber post to dentin at different parts of the root.

**Materials and Methods:**

This in-vitro experimental study was conducted on 20 single-rooted premolars. The teeth were decoronated at 1mm coronal to the cementoenamel junction (CEJ), and the roots underwent root canal treatment. Post space was prepared in the roots. Afterwards, the samples were randomly divided into two groups. In group 1, the fiber posts were cemented using Rely X Unicem cement, while in group 2, the fiber posts were cemented using Duo-Link cement, according to the manufacturer’s instructions. The intracanal post in each root was sectioned into three segments of coronal, middle, and apical, and each cross-section was subjected to push-out bond strength test at a crosshead speed of 1mm/minute until failure. Push-out bond strength data were analyzed using independent t-test and repeated measures ANOVA.

**Results:**

The bond strength at the middle and coronal segments in separate etch adhesive cement group was higher than that in self-adhesive cement group. However, the bond strength at the apical segment was higher in self-adhesive cement group compared to that in the other group. Overall, the bond strength in separate etch adhesive cement group was significantly higher than that in self-adhesive cement group (P<0.001).

**Conclusions:**

Bond strength of fiber post to intracanal dentin is higher after the use of separate etch adhesive cement compared to self-adhesive cement.

## INTRODUCTION

Intracanal posts were first introduced in the 17^th^ century to provide adequate retention for restoration of endodontically treated teeth at risk of fracture in function. Intracanal posts are available in different types of metal, ceramic and fiber posts. Non-metal posts are increasingly used due to high demand for esthetics and recent advances in adhesive systems [1–3]. Composite posts are among the most commonly used non-metal posts, and are composed of carbon, silica and quartz fibers embedded in epoxy resin matrix. In 1990, reinforced fiber posts containing quartz and glass fibers were introduced to the market. Elasticity modulus close to that of dentin, the ability to bond to intracanal dentin, and conservative tooth preparation are among the advantages of fiber posts [4,5]. These posts have stress distribution pattern similar to that of natural teeth, and minimize the risk of root fracture. Moreover, fractures caused due to the application of fiber posts have higher reparability compared to those caused by cast posts [6,7]. Bonding of fiber posts to intracanal dentin is challenging. Several factors affect the retention of fiber posts in root canals, such as the type of the tooth, root canal treatment, intracanal post surface preparation, bonding agent and cement, and method of application of the cement [8]. Despite the advantages, some failures have been witnessed after the use of fiber posts, such as root fracture, core fracture, and debonding [9]. Complete debonding is the most favorable mode of failure [10]. Partial debonding at the coronal section of the root would cause leakage and secondary caries. Partial debonding, especially at the apical area, interferes with stress distribution along the root, and may increase the risk of root fracture [11]. It has been reported that cementation of posts with adhesive systems increases the retention of the post, and decreases debonding and microleakage at the dentin-fiber post interface [12,13]. Separate etch adhesives and self-adhesives are among the most commonly used adhesives for this purpose. In separate etch adhesive system, acid and bonding agent are used separately. In self-adhesive systems, acid and bonding agent are used simultaneously without rinsing. Thus, the latter system is simpler, and less technique-sensitive [14]. Moreover, the risks of over-drying or excess moisture, and their adverse effects on the bond strength are eliminated due to decreased steps of the bonding procedure [15]. Dentists should choose the type of cement and post based on clinical indication, required bond strength, predictability, and long-term success, instead of simplicity, or to avoid technique missteps which would compromise the restoration. Thus, this study sought to compare the effects of self-adhesive and separate etch adhesive dual cure resin cements on the bond strength of quartz fiber post to dentin at different parts of the root.

## MATERIALS AND METHODS

In this in-vitro experimental study, 20 freshly extracted single-canal human premolars with complete apices were used, which had no fracture or defect in the dentin. The teeth were cleaned in an ultrasonic bath, and were stored in distilled water and 1% thymol solution for two weeks. All the teeth were decoronated at 1mm coronal to the cemento-enamel junction (CEJ) of the proximal surface, perpendicular to the longitudinal axis of the tooth, using a diamond disc and high-speed handpiece under water coolant. Working length was determined 1mm short of the apex. The root canals were cleaned and shaped using K-Flex files (Maillefer, Ecublens, Switzerland) via the step back technique. The root canals were rinsed with sodium hypochlorite. All the root canals were cleaned to #30 apical file, and were shaped conically to #70 file. The canals were then filled with gutta percha (Maillefer, Ecublens, Switzerland), and AH26 sealer (Dentsply Maillefer, Ballaigues, Switzerland) using lateral compaction technique. The teeth were then stored in saline at 37°C for one week in order for polymerization to complete [16]. The selected post was quartz fiber post (#2, Matchpost, Merge, Paris, France). Post space was prepared using #2 and #3 Gates Glidden drills (Maillefer, Ecublens, Switzerland) with 12mm length, and then was finished by using the post drills provided by the company. Afterwards, the teeth were randomly divided into two groups. In group 1, self-adhesive cement (Rely X Unicem, 3M ESPE, St. Paul, MN, USA), and in group 2, separate etch adhesive cement (Duo-Link cement; Bisco Dental, Schaumburg, IL, USA) were used. In group 1, the posts were cleaned by alcohol, and were air-dried. The root canals were rinsed and dried with paper cones. Next, the cement was mixed according to the manufacturer’s instructions, and the posts were dipped in the cement, and were placed inside the canals with firm finger pressure. The excess cement was removed. The posts were kept steady in the canals by the operator’s hand during cement setting.

**Fig. 1:**
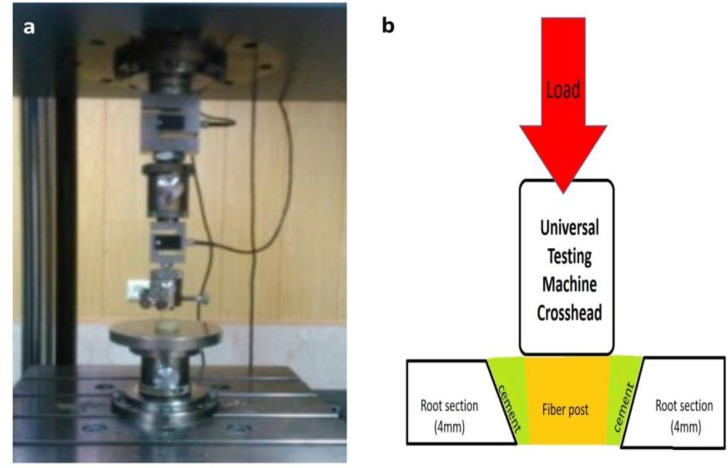
(a) Universal testing machine. (b) Schematic view of push-out bond strength test

In group 2, the posts were cleaned by alcohol, and were air-dried. Next, a drop of Z primer plus (Bisco Dental, Schaumburg, IL, USA) was applied to the post surface and dried for 3–5 seconds. The root canals were etched with 37% phosphoric acid for 15 seconds, and were rinsed. The excess water was eliminated by paper points. All Bond 2 (Bisco Dental, Schaumburg, IL, USA) was applied to the canal using a microbrush, and the excess adhesive was removed. Light curing was performed according to the manufacturer’s instructions for 10 seconds. Duo-Link cement was mixed according to the manufacturer’s instructions, and was injected into the canals by using a root canal syringe tip. The posts were coated with the cement, and were placed inside the canal with firm pressure, and were kept steady for 30 seconds. The excess cement was removed. Light curing was performed according to the manufacturer’s instructions for 40 seconds, while keeping the post in place by the operator’s hand. The intracanal posts (12mm in height) in both groups were marked and sectioned into three equal segments (4mm in length) of coronal, middle and apical using a diamond disc. Each segment was subjected to push-out bond strength test in a universal testing machine (Zwick Roell, Ulm, Germany), at a crosshead speed of 1mm/minute until debonding (separation of the post from the intracanal dentin). Bond strength was measured at the three segments in each sample (Fig. 1) [16].Data were analyzed with SPSS (statistical package for social sciences) version 22. Normality of data was proved according to Kolmogorov-Smirnov analysis. Repeated measures ANOVA was used to compare the bond strength at the three segments in each group. The bond strength at the three segments of the two groups was analyzed using independent t-test. P-value less than 0.05 was considered as significant.

## RESULTS

Independent t-test showed a significant difference in the bond strength of different root segments and, also between the two groups (Table 1). The bond strength at the coronal and middle segments of separate etch adhesive cement group was higher than that in self-adhesive cement group.

**Table 1.** The push-out bond strength of different root segments of the studied groups

**Root segment**	**Cement type**	**Mean (MPa)**	**Standard deviation**	**Minimum (MPa)**	**Maximum (MPa)**
**Coronal**	self-adhesive	5.7	1.03	4.64	7.43
	separate etch adhesive	14.0	2.61	11.02	17.39
**Middle**	self-adhesive	7.2	1.73	4.12	10.22
	separate etch adhesive	10.9	1.83	8.27	14.6
**Apical**	self-adhesive	9.6	1.07	8.1	10.89
	separate etch adhesive	7.0	1.50	5.31	9.75

However, the bond strength at the apical segment of self-adhesive cement group was higher than that in separate etch adhesive cement group. Overall, the bond strength in separate etch adhesive group was higher than that in self-adhesive cement group (P<0.001). Repeated measures ANOVA was used to compare the bond strength at the three segments in each group, and revealed that the bond strength increased from the coronal segment towards the apical part in self-adhesive cement group, while it increased from the apical part towards the coronal segment in separate etch adhesive group (P<0.001).

## DISCUSSION

This study assessed the push-out bond strength between fiber post and intracanal dentin at coronal, middle and apical parts of the root, using self-adhesive and separate etch adhesive cements. Several tests are used for assessment of the retention and bond strength of intracanal post to dentin, such as shear, microtensile, pull-out and push-out bond strength tests [17]. In push-out bond strength test, the load is applied along the longitudinal interface of adhesive-dentin. This test is more practical than the others [18]; therefore, it was employed in our study. Based on the results, the mean bond strength in separate etch adhesive group was higher than that in self-adhesive group, which may be related to the bonding mechanism. The mechanism of bonding by Rely X cement is via micromechanical retention and chemical bonds to hydroxyapatite. According to the manufacturer, it replaces polyacrylic with functional monomers of 4-META and modified esters [19]. Duo-Link eliminates the thin smear layer on the dentin by phosphoric acid etchant, and provides greater micromechanical retention [16]. Weak bonding after the use of Rely X cement may be due to the fact that although the pH of the cement is very low upon mixing, it cannot demineralize dentinal tubules, and consequently, methacrylate phosphate esters cannot adequately penetrate into the partially dissolved smear layer. This creates a gap between the surfaces, and decreases the bond strength [20,21]. This finding was in agreement with the results of the studies by Kececi et al [22], Radovic et al [23], and Rathke et al [24]. Based on our results, the bond strength in separate etch adhesive group decreased from the coronal part towards the apical segment. Since the bonding mechanism in this group is based on micromechanical retention following elimination of smear layer and exposure of dentinal tubules, decreased bond strength from the coronal part towards the apical segment can be attributed to the decreased density and diameter of dentinal tubules [25]. Also, risk of presence of gutta percha remnants, residual sealer, coarse debris, and thick smear layer is higher in apical areas, since the end point of the post space is hard to clean [26]. Moreover, the curing light hardly reaches the apical area, and this negatively affects the efficacy of bonding agent and cement at the depth of the canal [27]. On the other hand, curing of cement in the apical region weakens the bond due to high C factor in the root and polymerization shrinkage stresses [19]. This finding was in line with the results of the study by Rathke et al [24]. In self-adhesive group of the present study, the bond strength increased from the coronal segment towards the apical region. Some studies have correlated the bond strength of self-adhesives to the density of the dentin; thus, they believe that bond strength increases in the apical region due to decreased density of tubules [28]. Moreover, high pressure is important for better adaptation of the cement to the walls; this is particularly important for parallel posts. Parallel posts were used in our study; thus, greater stress was created at the apical region during cementation [16]. Moreover, cement thickness increases from the apical part towards the coronal segment. It has been reported that the bond strength of self-adhesive cements decreases by an increase in cement thickness [29]. Thick cement layer presents a higher risk of voids and porosities, and experiences greater polymerization shrinkage [30]. This result was in accordance with those achieved by Giachetti et al [31], and Prado et al [32], and in contrast to those found by Wang et al [16]. In the study by Wang et al [16], the bond strength of self-adhesive cement decreased from the coronal part towards the apical region, which may be attributed to the use of conical intracanal posts.

Comparison of bond strength at different radicular cross-sections between the two groups showed that the bond strength at the apical region was higher in self-adhesive group compared to the same region in separate etch adhesive group. This is probably due to the higher moisture tolerance of self-adhesive cement, since water is formed during neutralization reaction of phosphoric acid methacrylate and hydroxyapatite-based fillers [19].

## CONCLUSION

Within the limitations of the present study, the results showed that the push-out bond strength between the fiber post and intracanal dentin at the coronal and middle regions was higher after using separate etch adhesive cement compared to self-adhesive cement.
